# Explanation for the Multi-Component Scintillation of Cerium Fluoride Through the Equilibrium and Photophysical Investigation of Cerium(III)-Fluoro Complexes

**DOI:** 10.3390/nano9101462

**Published:** 2019-10-15

**Authors:** Zsolt Valicsek, Máté Kovács, Ottó Horváth

**Affiliations:** 1 Department of General and Inorganic Chemistry, Faculty of Engineering, University of Pannonia, Egyetem u. 10, H-8200 Veszprém, Hungary; valicsek@almos.uni-pannon.hu; 2 Department of Analytical Chemistry, Faculty of Engineering, University of Pannonia, Egyetem u. 10, H-8200 Veszprém, Hungary; mate.kovacs@richter.hu

**Keywords:** cerium(III)-fluoro complexes, complex equilibria, spectrophotometry and spectrofluorometry, emission lifetime, multi-component decay

## Abstract

CeF_3_ displays favorable scintillation properties, which have been utilized for decades in various solid-state systems. Its emission undergoes multi-component decays, which were interpreted by lattice defects and so-called intrinsic features herein. This study of the complex equilibria in connection with photophysical behavior of the cerium(III)-fluoride system in solution gave us the possibility to reveal the individual contribution of the [Ce^III^F_x_(H_2_O)_9−x_]^3−x^ species to the photoluminescence. Spectrophotometry and spectrofluorometry (also in time-resolved mode) were used, and combined with sophisticated evaluation methods regarding both the complex equilibria and the kinetics of the photoinduced processes. The individual photophysical parameters of the [Ce^III^F_x_(H_2_O)_9−x_]^3−x^ complexes were determined. For the kinetic evaluation, three methods of various simplifications were applied and compared. The results indicated that the rates of some excited-state equilibrium processes were comparable to those of the emission decay steps. Our results also contribute to the explanation of the multi-component emission decays in the CeF_3_-containing scintillators, due to the various coordination environments of Ce^3+^, which can be affected by the excitation leading to the dissociation of the metal-ligand bonds.

## 1. Introduction

Rare-earth metal ions can serve as centers in efficiently fluorescent artificial complexes [1] or natural minerals [2]. Among lanthanide ions, cerium(III) has been extensively studied because the one electron in a 4f orbital offers a simple model system to understand the f-electronic structure. Its generally intensive, parity allowed absorptions in the UV region, originating from 4f-5d transitions, make it possible to detect the electronic changes during complexation at relatively low concentrations. Cerium(III)-doped or based materials are among the most usable and efficient scintillators, owing to their fast and intensive fluorescence [3,4,5,6,7,8,9,10,11]. CeF_3_ is one of the most investigated cerium(III) compounds because the fluoride ions can control the mobility of lanthanide ions in nature; besides, cerium(III) fluoride is less hygroscopic than, e.g., cerium(III) chloride.

The ultraviolet (visible) absorption spectrum of cerium(III) ions with a [Xe]4f^1^ electron configuration is determined by the spin and symmetry allowed 4f–5d transitions, instead of weak f-f absorptions characteristic of lanthanides. The spectrum is complicated because the 4f^1^ subshell is split by spin-orbit coupling about 2000 cm^−1^: ^2^F → ^2^F_5/2_ + ^2^F_7/2_.

The 5d subshell is occupied by electron excitation, and has a crystal-field splitting about 10,000 cm^−1^, depending on the type of the ligands and on the symmetry of the structure. CeCl_3_ salt solved in water produces [Ce^III^(H_2_O)_9_]^3+^ nonaaqua-cerium(III) complex with TPRS-9 (trigonal prism, square face tricapped) structure, in which the symmetry is D_3h_. Hence, the following split takes place: ^2^D → ^2^A + ^2^E_(1)_ + ^2^E_(2)_. Notably, in the single crystals of the early lanthanide ions, also containing the water of crystallization, the coordination number is nine, as in the case of Ce^3+^, whereas around the smaller lanthanides (of higher atomic number) only eight ligands can be coordinated [12].

The absorption bands of the cerium(III) complexes, especially those of lower energy, significantly depend on the solvent applied [13,14]. Besides, the excitation spectra are strongly affected by the relative position of the frontier orbitals of the metal center and the ligand (e.g., F^−^) [15]. The increasing softness of the halogeno ligands enhances the covalent character of the Ln-X bond as well as the intensity and the wavelength of the bands sensitive to this effect. The 4f → 5d electronic transition mixes with a Ce^3+^ 4f → X^−^ *n*d type, metal-to-ligand charge transfer (MLCT), resulting in a gradual redshift of the emission in the order of Cl–Br–I (362, 390 and 514 nm) [16].

In the case of CeF_3_, which is frequently applied for scintillators, two-component emission was detected with a 5-ns lifetime at 310 nm and a 30-ns lifetime at 340 nm, the ratio of which proved to be temperature-dependent [17]. According to an interpretation, one component is an intrinsic (normal) type of radiation, while the other (extrinsic) one originates from the cerium(III) ions located at the perturbed (“outer”) sites of the lattice, where defects or contaminants can be effective [18,19].

Another approach attributed the faster, shorter-wavelength emission to the Ce^3+^ ions located at regular lattice sites, and the slower, longer-wavelength luminescence to the metal ions located at perturbed lattice sites [20]. These observations and interpretations clearly indicate that the local environment or coordination sphere of a fluorescent center significantly affects its emission [21]. Complex formation is a typical phenomenon changing the coordination sphere. Notably, photophysical and photochemical properties of some water-soluble lanthanide(III) ions, involving Ce^3+^, can be utilized for photocatalytic applications in aqueous systems [22].

Complexation between cerium(III) and fluoride ions is fairly difficult to study because of the precipitation of CeF_3_ at relatively low concentrations. Hence, the separation (distinction) of the mono and difluoro species in that equilibrium requires very careful procedures. Accordingly, the stability constants of those fluoro complexes have been determined, so far indirectly, by the measure of the concentration of free ions; however, the reaction can be spectrophotometrically (directly) investigated by using both absorption and emission spectra. With careful analyses of the titration series (increasing the fluoride concentration at fixed cerium(III) concentration), the individual spectra of the complex species can be calculated. That way, the concentrations of the different [Ce^III^F_x_(H_2_O)_9−x_]^3−x^ species can be determined in such equilibrium systems. The main purpose of this study was to determine the individual photophysical properties of these cerium(III) fluoro complexes. The results may contribute to the interpretation of the multi-component luminescence of the CeF_3_-based or CeF_3_–doped materials which are utilized as scintillators.

## 2. Materials and Methods 

### 2.1. Reagents and Solutions

All reagents were of analytical grade. A fluoride standard solution (Teknolab, Kolbotn, Norway) was used for the experiments. Cerium(III) chloride and sodium perchlorate (to adjust the ionic strength to I = 0.1 M) were purchased from Sigma-Aldrich Ltd (Budapest, Hungary), and dissolved in deionized, double-distilled water purified with a Millipore Milli-Q system (Millipore Inc., Bedford, MA, USA). Measurements were carried out after one-day storage, at room temperature and atmospheric pressure. Elimination of oxygen was realized with argon bubbling and Schlenk technique. The applied concentrations were: 1.0 × 10^−3^ M CeCl_3_ and 0–3.16 × 10^−3^ M F^−^. Twelve solutions of various fluoride concentrations were prepared without precipitation. In two further solutions, with 5.26 and 26.3 mM F^−^ concentrations, the appearance of precipitate was detected. The natural pH of the solutions studied was ~4.8 (without application of any buffer).

### 2.2. Instruments and Procedures

The absorption spectra were recorded with a single-beam SPECORD S-100 (Analytic Jena AG, Jena, Germany) diode array spectrophotometer. A PerkinElmer LS 50-B (PerkinElmer Inc., Waltham, MA, USA) spectrofluorometer was applied to measure the fluorescence and excitation spectra, which were corrected with the sensitivity of detector. Spectrum analyses were carried out by fitting Gaussian (and Lorentzian) curves in MS Excel.

Molar absorption (as well as emission and excitation) spectra, along with the stability constants of cerium(III)-fluoro complexes were simultaneously determined (according to Equations (1)–(3)) by fitting the calculated absorbances to the measured ones, using a support program based upon Newton–Raphson iteration (PSEQUAD) [23].

(1) $${\mathrm{Ce}}^{3+}+{x\;F}^{-}\Leftrightarrow {\left[{\mathrm{CeF}}_{x}\right]}^{3-x}$$

(2) $$\beta_{x}=\frac{\left[{\left[{\mathrm{CeF}}_{x}\right]}^{3-x}\right]}{{[\mathrm{Ce}}^{3+}{][F}^{-}{]}^{x}}$$

(3) $$A_{\lambda }=l\sum_{x=1}^{n}\varepsilon_{x,\lambda }\beta_{x}\prod_{i=1}^{k}{[c}_{i}{]}^{\alpha_{\mathrm{xi}}}$$

where β_x_ designates the formation constant of the xth complex, according to the process described by Equation (1); A_λ_ and λ are the absorbance and the wavelength, respectively; l is the path length (cm); ε_x__λ_ is the molar absorption coefficient (M^−1^·cm^−1^) at λ wavelength; [c_i_] is the equilibrium concentration of the ith free analytes (Ce^3+^ and F^−^ in our systems); α_xi_ is its stoichiometric index in the corresponding reaction equation. Of course, [CeF_x_]^3−x^ in Equations (1) and (2) is the abbreviation of the [CeF_x_(H_2_O)_9−x_]^3−x^ species (x = 1–3), while Ce^3+^ designates [Ce(H_2_O)_9_]^3+^ in this case.

A matrix rank analyzer (MRA) software with Gauss-Jordan elimination steps [24] was applied for the determination of the number of the species absorbing in the spectrophotometric titrations.

Fluorescence quantum yields were determined by using naphthalene in cyclohexane as a reference with the quantum yield (Φ_r,ref_) of 0.23 at 258 nm excitation [25]. Quantum yields of the Ce(III) species (Φ_r,i_) were calculated from the relative intensities; i.e., the ratios of the integrated areas in the (corrected) titrimetric emission spectra and the reference emission spectrum (T_i_/T_ref_), taking the refractive indices of the applied solvents, n_i_ and n_ref_ into account also (according to Equation (4)). In our solutions, at a constant Ce(III) concentration, the excitation at the quasi-isosbestic point of the absorption spectra of the cerium(III) complexes ensured that the number of photons absorbed by the Ce(III) species was practically the same, independent of the actual molar fractions. Additionally, the reference system had the same absorbance at the excitation wavelength.

(4) $$\Phi_{r,i}=\Phi_{r,\mathrm{ref}}\frac{T_{i}}{T_{\mathrm{ref}}}\;{\left(\frac{n_{i}}{n_{\mathrm{ref}}}\right)}^{2}$$

The amount of light absorbed by the individual cerium(III) complexes were proportional to their actual molar fractions.

The fourth overtone (266 nm, 3.5 ns halfwidth and 10 mJ energy) of a Quantel Brilliant Nd-YAG laser (Quantel Laser, Les Ulis, France) with a Tektronix TDS 684 A (1 GHz, 5 GSample) digital oscilloscope (Tektronix Inc., Beaverton, OR, USA) was applied to determine the luminescence lifetime [26] of the cerium(III)-fluoro complexes, which was near to the laser halfwidth. Hence, a deconvolution method [27] was used for the calculation of the luminescence lifetime, τ. In this method, three parameters (τ, θ_I_ and Δt_trigger_) were fit, according to Equations (5) and (6).

(5) $$I^{\lambda }\left(t\prime \right)=\theta_{I}\sum_{i=0}^{t_{\mathrm{laser}}}I_{\mathrm{laser}}^{\lambda }\left(i\right)\;\exp(-\frac{{t\prime -t}_{\mathrm{laser}}}{\tau })$$

(6) $$t\prime =t_{\mathrm{measured}}-{\Delta t}_{\mathrm{trigger}}$$

where Δt_trigger_ is the trigger delay (which has an uncertainty for the individual flashes), and θ_I_ is the normalizing factor adjusting the same intensity maximum for each emission decay.

From the luminescence lifetime and the fluorescence quantum yield, the rate constant of the emission decay (k_d_ = k_r_ + k_nr_) can be resolved to the rate constants of the radiative (k_r_) and non-radiative (k_nr_) processes, according to Equation (7).

(7) $$\Phi_{r}=\frac{k_{r}}{k_{r}+k_{\mathrm{nr}}}=k_{r}\tau$$

For the kinetic evaluation of the experimental data (i.e., the emission decay functions of the excited-state complexes) obtained regarding each sample with various ratios of the [Ce^III^F_x_(H_2_O)_9−x_]^3−x^ species, three different methods were applied.

(1) Supposing that the decay processes are significantly faster than the equilibrium reactions between the *[Ce^III^F_x_(H_2_O)_9__−x_]^3^^−x^ complexes, i.e., the complex formation and dissociation in the excited state are neglected, the measured emission decay curves were considered as the sum of the first-order decays of the individual (excited-state) Ce(III) species. Based on this approach, the individual fluorescence lifetimes of these complexes could be determined.

(2) In another simplified approach, the equilibrium reactions between the *[Ce^III^F_x_(H_2_O)_9__−x_]^3^^−x^ complexes were supposed to be considerably faster than the decay processes [28], according to Equations (8) and (9), where Φ(i) is the partial molar fraction of the ith complex species (I = 0–3). Due to the fast equilibrium processes, the individual excited species jointly decay; i.e., with the same rate. Hence, this decay can be described with a single-exponential function for each sample (i.e., for each complex equilibrium at the actual ligand (fluoride) concentration). The observed emission lifetime for a sample is considered as the weighted average of the individual lifetimes of the complex species, where the weights are the corresponding partial mole fractions (Φ(i)). The same is valid for the decay and radiation rate constants.

(8) $$\frac{1}{\tau_{\mathrm{obs}}}=\sum_{i=0}^{n}\frac{\varphi_{i}}{\tau_{i}}$$

(9) $$k_{r,\mathrm{obs}}=\frac{\Phi_{r,\mathrm{obs}}}{\tau_{\mathrm{obs}}}=\sum_{i=0}^{n}\varphi_{i}\times k_{r,i}$$

Additionally, in this case, the effects of the excitation on the equilibrium processes were neglected; i.e., rate constants for the complex formation and dissociation were considered to be the same in the excited state as in the ground state.

(3) If comparable decay and equilibrium rate constants for the excited-state Ce(III) species were supposed, the solution of a rather complicated differential equation system was realized by using a special kinetic program, called ZITA [29]. It was based on a Gauss–Newton–Marquardt method, giving numeric solutions for almost any kind of kinetic problem (from enough measured data). Namely, the rate constants of the excited-state equilibrium processes could be calculated. Hence, the reliabilities of the other two (simplified) methods could be compared.

## 3. Results and Discussion

### 3.1. Absorption Spectra and Equilibria

**Figure 1:**
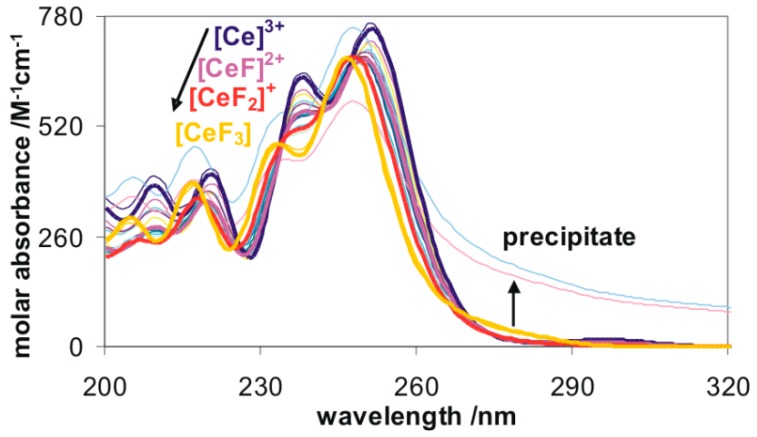
The series of titrimetric absorption spectra of 1.0 mM CeCl_3_ with fluoride ions in the 0–3.16 mM concentration range without the appearance of precipitate, and at 5.26 and 26.3 mM F^−^ concentration, which resulted in precipitation. The calculated individual molar absorption spectra are represented by the thick lines. [CeF_x_(H_2_O)_9−x_]^3−x^ is abbreviated as [CeF_x_]^3−x^.

**Figure 2:**
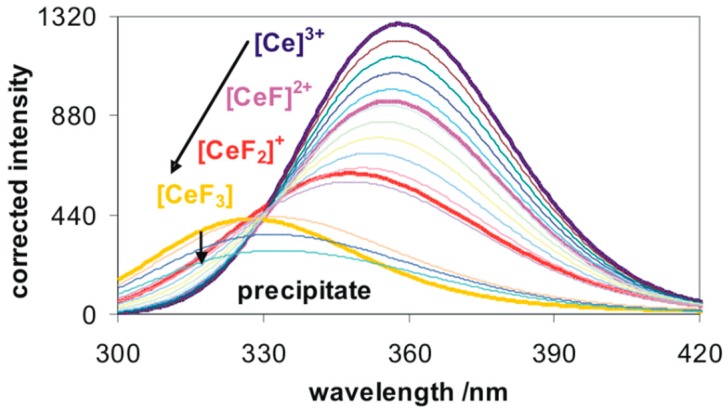
Emission spectra corresponding to the absorption spectra on Figure 1. Excitation wavelength is one of the quasi isosbestic points at 248 nm. The calculated individual fluorescence spectra are represented by the thick lines. [CeF_x_(H_2_O)_9−x_]^3−x^ is abbreviated as [CeF_x_]^3−x^.

The evaluation of the series of 12 spectra (Figure 1), by using Equations (1)–(3), PSEQUAD and MRA (see Section 2.2), unambiguously indicated that four species were responsible for the spectral changes; i.e., the colloidal cerium(III) fluoride behaved spectrophotometrically as a complex in solution, due to its very small size. The precipitation could only be detected (with the rising of the baseline in absorption spectra (Figure 1), as well as the decrease of the luminescence intensity (see later in Figure 2)) at significantly higher fluoride concentrations, due to the formation of larger nanoparticles [30]. Figure 1 also displays the individual spectra of the different Ce(III) species. Table 1 summarizes the complex formation constants and the absorption data of the individual species, obtained from the spectrum analyses by fitting Gaussian (and Lorentzian) curves (see Figure S1 in the Supplementary Materials (SM)).

Stability constants of cerium(III)-fluoro complexes have so far been indirectly determined by the measure of the concentrations of free ions with potentiometric, radiometric, solubility, ion-change, liquid–liquid extractive, polarographic, calorimetric, coulometric, electronic migration, distributive and spectrophotometric methods. However, in our study the pieces of information about the different fluoro complexes are direct. Hence, the description of these equilibria should be more accurate. Our stability constants, transformed from their original values (β_x_) to those for the reaction between cerium(III) ion and hydrogen fluoride (*β_x_ = β_x_/β_HF_^x^), are close to the data found in the literature, lg*β_1_ = 2.46–3.29 and lg*β_2_ = 4.60–6.57 (depending also on the ionic strength) [31,32,33,34]. Generally, these kinds of equilibrium constants (i.e., *β_x_) are given in the literature because the applied lower pH results in the protonation of fluoride to hinder the hydrolysis of cerium(III) ion.

In the formation of fluoro complexes, fluoride ions replace, consecutively, the initial water ligands in the TPRS-9 structure, [Ce^III^(H_2_O)_9_]^3+^. The symmetry is reduced in the first and second coordination steps, but the type of the split does not change (Figure 1), only its measure (last row in Table 1). As a consequence of the coordination of the third fluoro ligand, the D_3h_ symmetry is recovered in the transitionally forming [Ce^III^F_3_(H_2_O)_6_] complexes (the trigonal prism on the square face is tricapped by the three fluorides), which can swiftly agglomerate, excluding the water molecules from the coordination sphere of metal ion. Nevertheless, the CeF_3_ crystallizes in TPRS-9 geometry as well, thus the symmetry is not modified further. The energy gap between the two lowest terms in electron-excited state (^2^A and ^2^E_(1)_) is significantly decreased compared to the initial species, from 6000 to 2800 cm^−1^, because the energy level of the ^2^A term (which can originate from the 5d_z2_ orbital) rises much more than that of the other two terms. The reason for this phenomenon may be that the fluorides in the solid CeF_3_ are located closer to the z-axis, and perturb the 5d_z2_ orbital harder than the water molecules do in the aqua complexes. The crystal-field splitting decreases slightly in the mono and difluorocerium(III) species, but drastically in the trifluoro complex, compared to [Ce^III^(H_2_O)_9_]^3+^. This observation is in accordance with the relative position of the two ligand-types to each other in the spectrochemical series. It is manifested in the gradually increasing blueshift of the absorption (Figure 1 and Table 1) and emission bands (see later in Figure 2 and Table 2) during the complexation.

### 3.2. Emission and Excitation Spectra

The appearance of quasi isosbestic points in the absorption spectra (Figure 1) made it possible to follow the complexation in the luminescence spectra, also in quantity, resulting in the determination of the nearly exact relative emission intensities of the individual [Ce^III^F_x_(H_2_O)_9−x_]^3−x^ species (Figure 2 and Table 2).

Moreover, the emission bands of the fluoro complexes show blueshifts compared to that of [Ce(H_2_O)_9_]^3+^, but their measures deviate from those of the absorption bands because the ground and excited electronic states are modified by the complexation in different ways. The quantitative data of this structural difference between the ground and the corresponding excited states is the Stokes-shift, which is highest for [CeF_3_(H_2_O)_6_] (Table 1).

The fluorescence quantum yields decrease by the increase of the number of the fluoro ligands, along with the increase of the λ_1_ excitation energies (Table 1) and the corresponding Stokes-shifts. Dissolved oxygen has no effect on these emissions. The explanation of this phenomenon is that the corresponding transitions take place between the doublet states of the Ce^3+^ ion, and the triplet → singlet quenching process of O_2_ does not interact with these transitions.

The fluorescence wavelengths and the Stokes-shifts for CeCl_3_ and CeF_3_ were determined earlier, but those for the mono and difluoro species are so far unknown. The emission quantum yield for CeCl_3_ was found to be close to unity: 0.99 ± 0.03 [17], which was reproduced in our experiments.

**Figure 3:**
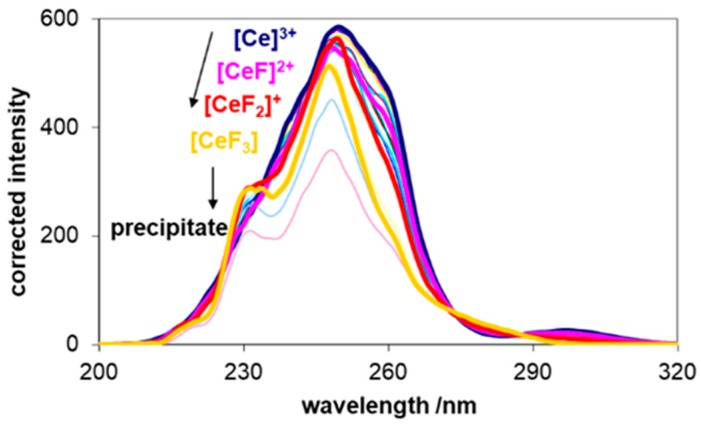
Individual excitation spectra of the Ce(III) species. [CeF_x_(H_2_O)_9−x_]^3−x^ is abbreviated as [CeF_x_]^3−x^.

The appearance of a quasi isostilbic point in the series of the emission spectra (at about 330 nm, Figure 2) resulted in two further experimental possibilities: (1) the simultaneous determination of the so-far-unidentified fluorescence lifetimes of the cerium(III)-fluoro complexes by deconvolution using Equations (5) and (6) (see in Section 3.3); (2) the determination of the individual excitation spectra (with nearly the same relative intensities for the different Ce(III) species; Figure 3). Notably, due to the small overlap of the individual excitation and the corresponding fluorescence spectra, i.e., the large Stokes-shifts, the inner absorptions of the emitted radiations were negligible in these cases.

Hypsochromic effects also dominate in the series of the excitation spectra during the complexation. Nevertheless, the individual excitation spectra of the Ce(III) species are not totally identical to the corresponding absorption ones, as indicated in Table S1. The main differences originate from the fact that the λ_5_ absorptions are ineffective for excitation in all species; however, the λ_4_ absorptions are slightly efficient in the case of di and trifluorocerium(III). The reason for this phenomenon may be the weak coupling between the ^2^E_(2)_ and ^2^E_(1)_ terms, whose connection will be slightly stronger, due to the coordination of two or three fluorides to the cerium(III) ion. Besides, a new band appears or becomes more intensive in the excitation spectra at about 260 nm for all Ce(III) species in this system.

**Scheme 1:**
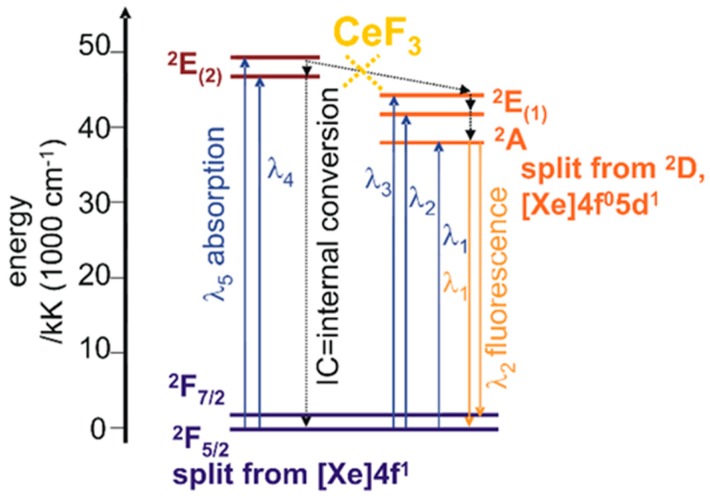
Simplified Jablonski diagram of CeF_3_.

On the basis of the analyses of the individual absorption, for emission (intensity and decay—see later in Section 3.3) and excitation spectra, the electronic transitions involved in these photoluminescence processes can be reliably assigned. The photophysical properties characterized can be represented by the Jablonski diagram of CeF_3_ (Scheme 1).

### 3.3. The Decays of Excited States

**Figure 4:**
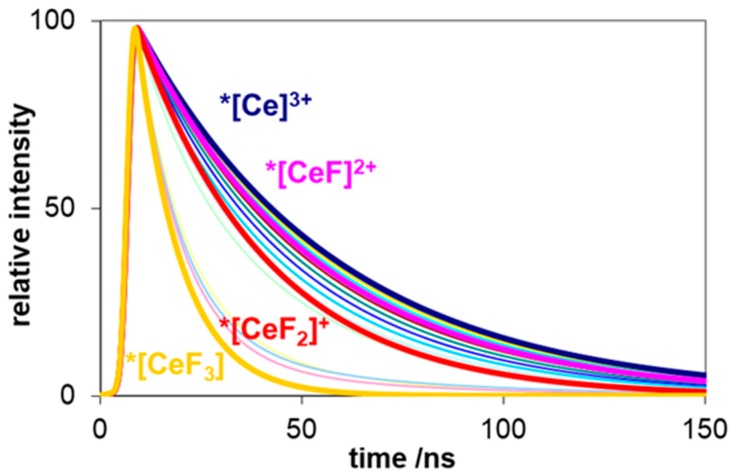
Fluorescence decay curves recorded at 330 nm—the quasi isostilbic point in Figure 2. The individual decay curves determined for the excited-state Ce(III) species are represented by the thick lines. *[CeF_x_(H_2_O)_9−x_]^3−x^ is abbreviated as *[CeF_x_]^3−x^.

The appearance of a quasi isostilbic point in the series of emission spectra (at about 330 nm) resulted also in the possibility of simultaneous determination of the so-far-unknown individual fluorescence lifetimes (and the corresponding decay curves) of the cerium(III)-fluoro complexes by deconvolution of the experimental data, using Equations (5) and (6). The normalized decay curves are shown in Figure 4.

**Scheme 2:**
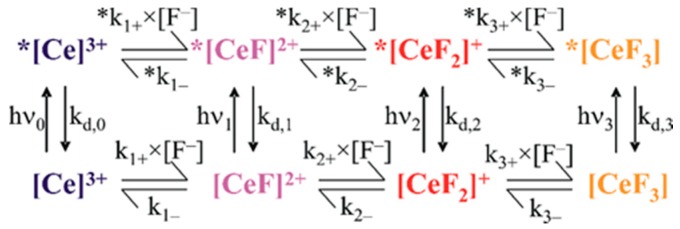
Rate constants for the various reactions of the cerium(III) species in the presence of fluoride ions in both ground and excited states. [CeF_x_(H_2_O)_9−x_]^3−x^ is abbreviated as [CeF_x_]^3−x^.

The kinetic processes taking place with the species (in both ground and excited states) of this system, along with the corresponding rate constants, are summarized in Scheme 2, where an asterisk designates the excited-state species and the rate constants for their reactions.

Since the samples were excited close to a quasi-isosbestic point (248 nm, where the molar absorbances of the different Ce(III) species are approximately equal), the light absorption of a species was proportional to its partial molar fraction. These fractions were determined by the ground-state equilibria of the fluoro complexes.

In the two simplified kinetic evaluation methods described in Section 2.2, the formation and the dissociation rate constants were considered to be equal in the ground and the excited states (k_i+_ = *k_i+_ and k_i−_ = *k_i−_). Hence, their ratios; i.e., the stability constants were the same in both states (K_i_ = *K_i_). In the first simplified approach, these rate constants were supposed to be many (at least five orders of magnitude) lower than those of the decays (k_d,i_) (slower equilibrium processes), while a reversed relationship was assumed in the second approach (faster equilibrium processes).

In the case of the first method, the excited-state lifetimes of the four Ce(III) species (τ_i_) could be determined by deconvolution based on Equations (5) and (6). The individual fluorescence intensities were calculated on the basis of the actual molar ratios of the complexes and the fluorescence quantum yields determined under steady-state circumstances. The photophysical parameters determined for each species are summarized in Table S2.

In the second simplified approach, τ_obs_ (i.e., a single-exponential decay function) could also be determined on the basis of Equations (5) and (6). In this case, however, the decay rate constants (k_d,i_ and k_r,i_) were calculated by taking the quantum yields measured for each equilibrium. The rate constants for each equilibrium were the weighted averages of the individual ones, where the weights were the partial mole fractions, according to Equations (8) and (9). The results obtained are summarized in Table S3).

Notably, the simplifications were based on the assumptions that the rates of the emission decays and the equilibrium processes of the excited-state complexes deviated by several orders of magnitude; accordingly, they took place on quite different time scales, offering the possibility of the separation of the decay and the equilibrium processes. However, the results clearly showed that these simplified approaches could not adequately describe the kinetics of the reactions in our systems.

Interestingly, both simplified methods provided very similar values for the [Ce(H_2_O)_9_]^3+^ and [CeF(H_2_O)_8_]^2+^ species, while for the di and trifluoro complexes, the decays were significantly slower in the second approach. These deviations indicated that the equilibrium relations could significantly change in the excited state. This was checked by the application of a sophisticated kinetic evaluation program (ZITA, see in Section 2.2). It could fit all rate constants for the excited-state species in Scheme 2, utilizing all the 12 decay curves recorded for the samples of various compositions. This method could be fully applied when comparable decay and equilibrium rate constants were assumed. The program was used for the simplified models, too; however, in those cases the values of several rate constants were correlated. Besides, some rate constants of the equilibrium processes were extremely high or low, which was unrealistic. The rate constants for the excited-state species, obtained with the simplified methods, are summarized in Tables S4 and S5, along with a short discussion in the SM.

**Figure 5:**
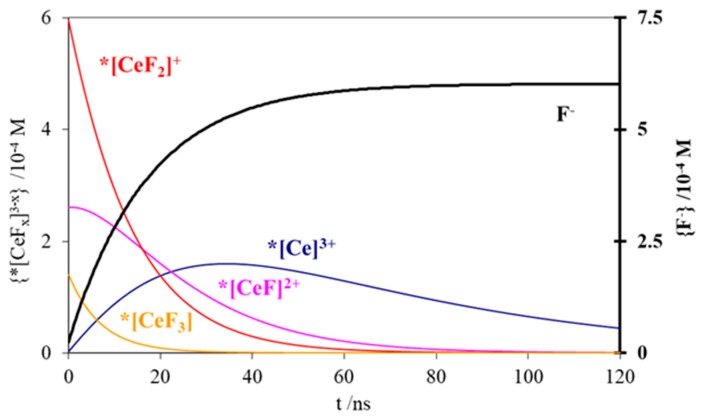
The change of the concentration of each species in the system (c(Ce^3+^) = 1.0 mM and c(F^−^) = 1.9 mM) during the 120 ns period after the laser excitation, due to the evaluation method assuming comparable decay and equilibrium rate constants. *[CeF_x_(H_2_O)_9−x_]^3−x^ is abbreviated as *[CeF_x_]^3−x^.

The best solution (with the best fitting to the measured decay curves) was given by the method supposing comparable decay and equilibrium rate constants. As the values in Table 3 indicate, the dissociation rate constants for the complexes in the excited state are comparable to the decay ones. Hence, the main cause of the multi-exponential emission decay in the cerium(III)-fluoride system is the increase of the dissociation of the fluoro complexes in the excited state—finally producing free (i.e., equated) Ce^3+^ ions ([Ce(H_2_O)_9_]^3+^). At the same time, the complex formations (in the excited state) become of minor significance; i.e., photoinduced dissociations take place. Therefore, the concentration of the free fluoride ions increases. These processes are clearly demonstrated in Figure 5, displaying the change of the concentrations of each species in the system during the 120-ns period right after the laser pulse.

The corresponding concentration versus time functions were also calculated for the simplified approaches (Figure S2). They dramatically differ from those in Figure 5, which is discussed in the SM.

The comparison of the results provided by the three evaluation methods unambiguously indicated that the approach assuming comparable decay and equilibrium rate constants proved to be the most reliable. In this case, however, the kinetic evaluation program delivered only the decay rate constants (k_d,i_) from the photophysical parameters. These data were closer to the values provided by the method supposing slower equilibrium processes (Table S4) than those obtained by the other simplified method (Table S5). Besides, the latter approach proved to be the worst in several respects for the description of the system studied. Hence, for the calculation of further photophysical parameters in the case of the comparable rate constants, the way they were applied in the method of slower equilibria was utilized; i.e., further calculations started with the emission quantum yields obtained from the steady-state irradiation experiments. Table 4 summarizes the photophysical data gained in this procedure.

The “pure” fluorescence lifetime (τ_0_ = 1/k_r_) indicates the lifetime of the excited state if it could only decay through a radiation process. In the case of the values obtained by the two simplified methods (Tables S2 and S3), the coordination of the first two fluoro ligands moderately increased, while that of the third one considerably decreased τ_0_. According to the more reasonable values provided by the third method, τ_0_ gradually decreases upon increasing the number of the fluoro ligands. Besides, the real emission lifetime (τ_i_) and the quantum yield (Φ_r,i_) also display similar tendencies, due to the significant increase of the non-radiative decay rate (k_nr,i_). In other words, the complexation speeds up the non-radiative decay, which becomes the determining factor in the decrease of τ_i_. The radiative decay just slightly accelerates as a consequence of the coordination of fluoride ions, hence its contribution to the faster decay is of minor significance (Table 4).

Comparisons with the data in the literature can only be made in the case of the radiation lifetimes. Our value for [Ce(H_2_O)_9_]^3+^ is in accordance with that determined in aqueous solution (44 ns [35]). Numerous values were published for solid CeF_3_: 20 ns in the case of optical excitation [36]; 30 ns in scintillation [37]; and with two-component decays, 3 and 27 ns [38], 5 and 30 ns [17] and 17 and 29 ns [39]. When CeF_3_ was used as the dopant in scintillators, 30 ns was measured in glass [40], and multi-exponential decays were observed in those cases: 1.9 and 38 ns [41]; 17 and 40 ns [20]; and 2–10 and 15–30 ns [18], along with 20, 330 and 2200 ns [42]. Except for the latter ones, our results are in accordance even with these solid-state lifetimes. Hence, they can be utilized for interpretation of the two or multi-component emission decays in scintillators.

The explanation in [20] is partly confirmed by our results, according to which the crystal defects can result in the movement of the anions into those defects, causing the distortion of the crystal field, hence the change of the photophysical features. If the coordination sphere or environment of the Ce^3+^ ions varies inside a crystal lattice, different complexes can be formed locally, with deviating photophysical parameters. Not only can the ionizing radiations increase the number of such defects, leading to the appearance of a faster emission component, as described in [36], but the optical excitation, at the surface of the lattice, can result in some rearrangement of the ligands, increasing the rate constant of the non-radiative decay. These possibilities clearly demonstrate that our results obtained for the equilibria and photophysical properties of cerium(III)-fluoro complexes in aqueous solution can contribute to the explanation of the multi-component scintillation of CeF_3_.

## 4. Conclusions

In this work, stability constants, individual absorption and emission properties of cerium(III)-fluoro complexes were carefully determined by steady-state spectrophotometry and spectrofluorometry, as well as time-resolved emission measurements. Our results clearly indicated that, in the system studied, the rates of the decay and the equilibrium processes for the excited-state cerium(III) complexes are comparable. Accordingly, the method applied to an appropriate evaluation of their emission decay curves must take both types of processes into account. As to the reactions involved in the equilibria of the excited-state complexes, dissociations are very competitive with the decays. Besides, higher number of the fluoro ligands promotes faster decay of the emitting species, primarily due to the acceleration of the non-radiative relaxation. These results may contribute not only to the interpretation of the multi-component scintillation of CeF_3_, but also to the design of scintillators involving Ce^3+^ ions in various coordination environments.

## Figures and Tables

**Table 1:** Stability constants and absorption data of cerium(III)-fluoro complexes from spectrum analysis. [CeF_x_(H_2_O)_9−x_]^3−x^ is abbreviated as [CeF_x_]^3−x^.

Complex	[Ce]^3+^	[CeF]^2+^	[CeF_2_]^+^	[CeF_3_]
**lgβ_x_**	-	6.49	11.46	15.45
lg*β_x_	-	3.34	5.18	6.03
**λ_1_/nm**	296	294	(290)	265
**ε_1_/M^−1^cm^−1^**	16.5	10.1	(0.0)	82.4
**shift_1_/cm^−1^**	-	195	693	3890
**λ_2_/nm**	251	250	248	247
**ε_2_/M^−1^cm^−1^**	738	669	674	663
**shift_2_/cm^−1^**	-	242	610	754
**λ_3_/nm**	237	235	233	232
**ε_3_/M^−1^cm^−1^**	523	391	312	420
**shift_3_/cm^−1^**	-	195	622	848
**λ_4_/nm**	221	220	218	217
**ε_4_/M^−1^cm^−1^**	390	331	341	380
**shift_4_/cm^−1^**	-	169	519	780
**λ_5_/nm**	210	209	206	206
**ε_5_/M^−1^cm^−1^**	241	146	165	207
**shift_5_/cm^−1^**	-	153	831	972
**crystal-field splitting/cm^−1^**	13,818	13,776	(13,957)	10,900

**Table 2:** Fluorescence data of cerium(III)-fluoro complexes from spectrum analysis. [CeF_x_(H_2_O)_9−x_]^3−x^ is abbreviated as [CeF_x_]^3−x^.

Complex	[Ce]^3+^	[CeF]^2+^	[CeF_2_]^+^	[CeF_3_]
**λ_1_/nm**	358	356	348	327
**shift/cm^−1^**	-	175	829	2641
**Stokes/cm^−1^**	5872	5892	5736	7122
**Φ_r_**	0.990	0.769	0.604	0.377

**Table 3:** Rate constants for fluorescence decay and excited-state equilibrium processes in the cerium(III)-fluoride system, calculated by the method supposing equilibrium processes with rates comparable to the photophysical ones. Additionally, the excited-state stability constants are given. [CeF_x_(H_2_O)_9−x_]^3−x^ is abbreviated as [CeF_x_]^3−x^.

Complex	k_d,i_/10^7^ s^−1^	*k_i+_/10^7^ M^−1^ s^−1^	*k_i−_/10^7^ s^−1^	lg*K_i_/M^−1^
**[Ce]^3+^**	1.98	-	-	-
**[CeF]^2+^**	2.83	0.348	3.80	−1.04
**[CeF_2_]^+^**	4.81	0.0915	3.04	−1.52
**[CeF_3_]**	9.84	120	4.43	1.43

**Table 4:** Photophysical parameters for the cerium(III)-fluoride system, calculated by the method supposing equilibrium processes with rates comparable with those of the photophysical ones. [CeF_x_(H_2_O)_9−x_]^3−x^ is abbreviated as [CeF_x_]^3−x^.

Complex	[Ce]^3+^	[CeF]^2+^	[CeF_2_]^+^	[CeF_3_]
**Φ_r_**	0.990	0.769	0.604	0.377
**τ/ns**	50.6	35.3	20.8	10.2
**k_r_/10^7^ s^−1^**	1.96	2.18	2.91	3.71
**k_nr_/10^7^ s^−1^**	0.0198	0.654	1.90	6.13
**τ_0_ = 1/k_r_/ns**	51.1	45.9	34.4	27.0
**k_d_ = 1/τ/10^7^ s^−1^**	1.98	2.83	4.81	9.84

## Supplementary Materials

The following are available online at https://www.mdpi.com/2079-4991/9/10/1462/s1: Figure S1: Analysis of the absorption spectrum of CeF_3_. Table S1: Excitation bands of cerium(III)-fluoro complexes compared to the absorption ones from spectrum analysis. Table S2: Photophysical parameters for the cerium(III)-fluoride system, calculated by the method supposing equilibrium processes much slower than the photophysical ones. Table S3: Photophysical parameters for the cerium(III)-fluoride system, calculated by the method supposing equilibrium processes much faster than the photophysical ones. Table S4: Rate constants for fluorescence decay and excited-state equilibrium processes in the cerium(III)-fluoride system, calculated by the method supposing equilibrium processes much slower than the photophysical ones. Additionally, the excited-state stability constants are given. Table S5: Rate constants for fluorescence decay and excited-state equilibrium processes in the cerium(III)-fluoride system, calculated by the method supposing equilibrium processes are much faster than the photophysical ones. Additionally, the excited-state stability constants are given. Figure S2: The change of the concentration of each species in the system (c(Ce^3+^) = 1.0 mM and c(F^−^) = 1.9 mM) during the 120-ns period after the laser excitation, due to the evaluation method assuming (a) slower or (b) faster equilibrium processes than the luminescence decay.
