# Supplementary material for: Explanation for the Multi-Component Scintillation of Cerium Fluoride Through the Equilibrium and Photophysical Investigation of Cerium(III)-Fluoro Complexes
Source: Nanomaterials (Basel). 2019 Oct 15;9(10):1462. doi: 10.3390/nano9101462 (PMC6835730; doi:10.3390/nano9101462)
Supplement: Supplementary file 1 [file nanomaterials-09-01462-s001.pdf]

Supplementary Information

for

Explanation for the Multi-Component Scintillation of Cerium Fluoride Through the Equilibrium and Photophysical Investigation of Cerium(III)-Fluoro Complexes

Zsolt Valicsek [1], Máté Kovács [2,†] and Ottó Horváth [1,*]

[1] Department of General and Inorganic Chemistry, Faculty of Engineering, University of Pannonia, Egyetem u. 10, H-8200 Veszprém, Hungary; valicsek@almos.uni-pannon.hu (Z.V.)

[2] Department of Analytical Chemistry, Faculty of Engineering, University of Pannonia, Egyetem u. 10, H-8200 Veszprém, Hungary; mate.kovacs@richter.hu (M.K.)

* Correspondence: horvath.otto@mk.uni-pannon.hu; Tel.: +36-88-624-000 / 6049 ext.

† Present affiliation: Gedeon Richter Plc. Research Analytical Department, Gyömrői út 19-21, H-1103 Budapest

---

Content **Page Nr.**

Content	Page Nr.
Figure S1, Tables S1-2	2
Tables S3-5	3
Figure S2	4

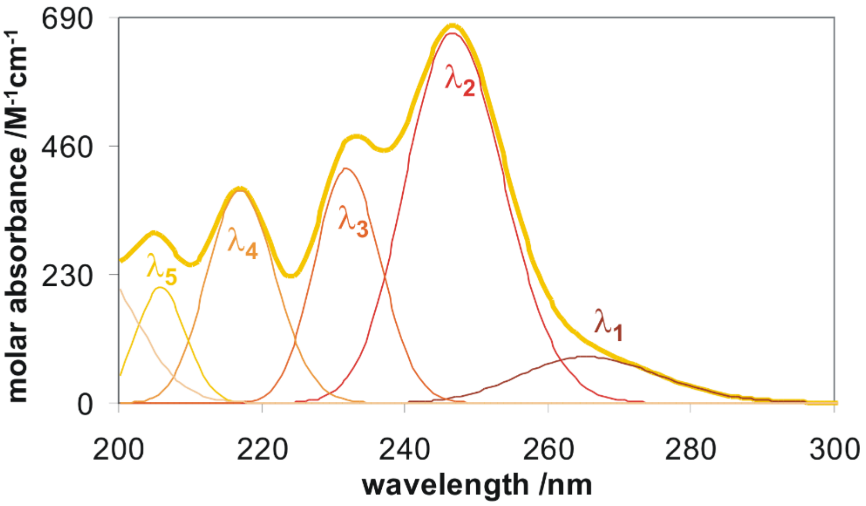

Figure S1. Analysis of the absorption spectrum of $[CeF_3(H_2O)_6]$.

Table S1. Excitation bands of cerium(III)-fluoro complexes compared to the absorption ones from spectrum analysis. $[CeF_x(H_2O)_{9-x}]^{3-x}$ is abbreviated as $[CeF_x]^{3-x}$.

**complex**		$[Ce]^{3+}$	$[CeF]^{2+}$	$[CeF_2]^{+}$	$[CeF_3]$
λ_1 /nm	**absorption**	296	294	290	265
	excitation	297	295	287	264
λ_2 /nm	**absorption**	251	250	248	247
	excitation	253	254	249	247
λ_3 /nm	**absorption**	237	235	233	232
	excitation	241	238	230	231
λ_4 /nm	**absorption**	221	220	218	217
	excitation	-	-	220	218
λ_5 /nm	**absorption**	210	209	206	206
	excitation	-	-	-	-

Table S2. Photophysical parameters for the cerium(III)-fluoride system, calculated by the method supposing equilibrium processes much slower than the photophysical ones. $[CeF_x(H_2O)_{9-x}]^{3-x}$ is abbreviated as $[CeF_x]^{3-x}$.

**complex**	$[Ce]^{3+}$	$[CeF]^{2+}$	$[CeF_2]^{+}$	$[CeF_3]$
Φ_r	0.987	0.784	0.658	0.387
τ /ns	49.0	44.5	36.6	13.8
k_r /10^7 s^{-1}	2.01	1.76	1.80	2.80
k_{nr} /10^7 s^{-1}	0.0255	0.486	0.934	4.44
$\tau_0=1/k_r$ /ns	49.7	56.7	55.6	35.7
$k_d=1/\tau$ /10^7 s^{-1}	2.04	2.25	2.73	7.24

Table S3. Photophysical parameters for the cerium(III)-fluoride system, calculated by the method supposing equilibrium processes much faster than the photophysical ones. $[CeF_x(H_2O)_{9-x}]^{3-x}$ is abbreviated as $[CeF_x]^{3-x}$.

complex	$[Ce]^{3+}$	$[CeF]^{2+}$	$[CeF_2]^+$	$[CeF_3]$
Φ_r	0.990	0.769	0.604	0.377
τ /ns	49.1	44.2	32.2	10.9
k_r /10^7 s^{-1}	2.02	1.74	1.88	3.45
k_{nr} /10^7 s^{-1}	0.0204	0.523	1.23	5.71
$\tau_0=1/k_r$ /ns	49.6	57.4	53.2	29.0
$k_d=1/\tau$ /10^7 s^{-1}	2.04	2.26	3.11	9.16

Table S4. Rate constants for fluorescence decay and excited-state equilibrium processes in the cerium(III)-fluoride system, calculated by the method supposing equilibrium processes much slower than the photophysical ones. Additionally, the excited-state stability constants are given. $[CeF_x(H_2O)_{9-x}]^{3-x}$ is abbreviated as $[CeF_x]^{3-x}$.

complex	$k_{d,i}$ /10^7 s^{-1}	$^*k_{i+}$ /10^7 $M^{-1}s^{-1}$	$^*k_{i-}$ /10^7 s^{-1}	lg $^*K_i$ /M^{-1}
$[Ce]^{3+}$	2.05	–	–	–
$[CeF]^{2+}$	2.21	0.0392	0.0000000754	5.72
$[CeF_2]^+$	5.58	0.000561	2.91	-3.71
$[CeF_3]$	9.79	2.11	0.0587	1.56

Table S5. Rate constants for fluorescence decay and excited-state equilibrium processes in the cerium(III)- fluoride system, calculated by the method supposing equilibrium processes are much faster than the photophysical ones. Additionally, the excited-state stability constants are given. $[CeF_x(H_2O)_{9-x}]^{3-x}$ is abbreviated as $[CeF_x]^{3-x}$.

complex	$k_{d,i}$ /10^7 s^{-1}	$^*k_{i+}$ /10^7 $M^{-1}s^{-1}$	$^*k_{i-}$ /10^7 s^{-1}	lg $^*K_i$ /M^{-1}
$[Ce]^{3+}$	2.09	–	–	–
$[CeF]^{2+}$	0.000741	67600000	94500	2.85
$[CeF_2]^+$	0.00560	21600	34.5	2.80
$[CeF_3]$	7.87	199000000	3130	4.80

Comparing to the ground-state situation, in the case of the assumption of slower equilibrium processes, the monofluoro cerium(III) complex will be more and more dominant in the luminescence because the di- and trifluoro complexes decay much faster (Fig. S2a), besides, $^*[CeF_2(H_2O)_7]^+$ is very dissociative (compared to the ground-state complex). The decay rate constants of $^*[Ce(H_2O)_9]^{3+}$ and $^*[CeF(H_2O)_8]^{2+}$ are very close, hence, their emission decay curves must be almost parallel, even if it cannot be seen in Fig. S2a because of the very low fraction of $^*[Ce(H_2O)_9]^{3+}$. Due to the slow equilibrium processes, the concentration of the free fluoride remains constant.

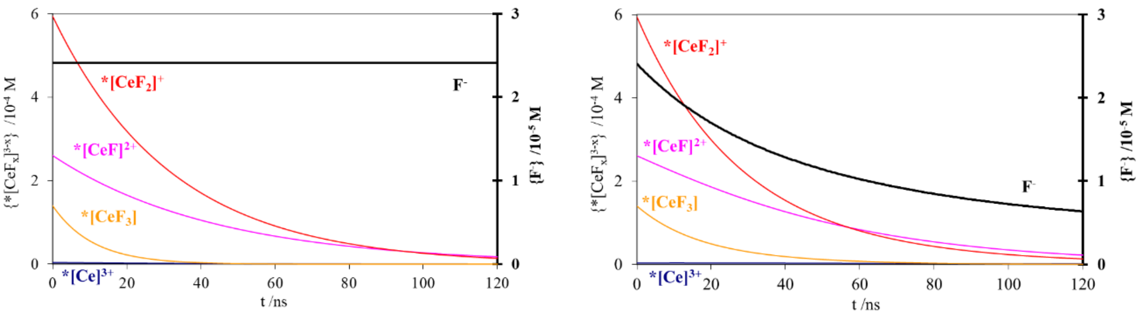

(a) **(b)**

Figure S2. The change of the concentration of each species in the system (c(Ce^{3+})=1.0 mM and c(F^-)=1.9 mM) during the 120-ns period after the laser excitation, due to the evaluation method assuming (a) slower or (b) faster equilibrium processes than the luminescence decay. $[CeF_x(H_2O)_{9-x}]^{3-x}$ is abbreviated as $[CeF_x]^{3-x}$.

In the case of the faster equilibrium processes, one may expect the same lifetime for each complex species, i.e., parallel decay curves. However, due to the stabilities of the excited-state complexes and the extremely high decay rate constants, $^*[CeF_2(H_2O)_7]^+$ disappears rather fast after the excitation (Fig. S2b), while the stability of the $^*[CeF_3(H_2O)_6]$ species increased (compared to the ground-state situation). Hence, its decay is much slower than that of $^*[CeF_2(H_2O)_7]^+$, due to the continuous supply from the excited-state complex equilibrium. Also in this case, the decay of $^*[CeF(H_2O)_8]^{2+}$ is the slowest one.
